# Long-Term Maintenance of Viable Adult Rat Sertoli Cells Able to Establish Testis Barrier Components and Function in Response to Androgens

**DOI:** 10.3390/cells10092405

**Published:** 2021-09-13

**Authors:** Hassan Kabbesh, Muhammad A. Riaz, Alexandra D. Jensen, Georgios Scheiner-Bobis, Lutz Konrad

**Affiliations:** 1 Center of Gynecology and Obstetrics, Faculty of Medicine, Justus-Liebig-University Giessen, Feulgenstr. 10-12, D-35392 Giessen, Germany; drhassankabbesh@gmail.com (H.K.); assad_riaz2k@yahoo.com (M.A.R.); 2 Center of Radiotherapy, Faculty of Medicine, Justus-Liebig-University Giessen, D-35393 Klinikstr, Germany; alexandra.jensen@uk-gm.de; 3 Institute for Veterinary Physiology and Biochemistry, School of Veterinary Medicine, Justus-Liebig-University Giessen, Frankfurter Str., D-35392 Giessen, Germany; georgios.scheiner-bobis@vetmed.uni-giessen.de

**Keywords:** Sertoli cells, blood testis barrier, tight junctions, androgen receptor, conditional reprogramming

## Abstract

A protocol for the isolation and long-term propagation of adult rat Sertoli cells (SCs) using conditional reprogramming (CR) was developed and the formation of tight junctions as an in vitro model for the blood testis barrier (BTB) was studied. Three pure primary SC lines were isolated successfully and maintained for several months without significant changes in expression levels of SC-typical markers such as SRY-box transcription factor 9 (SOX9), transferrin, clusterin, androgen receptor (AR), and GATA binding protein 1 (GATA1). In addition to AR expression, the tight junction proteins, zonula occludens-1 (ZO-1) and the junctional adhesion molecule-3 (JAM-3), were upregulated and the SC barrier integrity was enhanced by testosterone. Peritubular/myoid cells did not increase the tightness of the SC. The cytokines, interleukin-6 (IL-6), bone morphogenetic protein-2 (BMP2), and transforming growth factor beta-3 (TGF-β3), negatively affected the tightness of the SC barrier. We have established a protocol for the isolation and long-term propagation of highly pure primary adult rat SCs, which are able to respond to androgen treatments, to form tight junctions and to maintain the mRNA expression of SC-specific genes. By applying this new method, adult SCs can now be analyzed in more detail and might serve as an in vitro model for the study of many SC functions.

## 1. Introduction

Sertoli cells (SCs) are fundamental for studies of the blood testis barrier (BTB) and testicular immune privilege, as well as the analysis of spermatogenesis-associated events in vitro and in vivo [1]. Therefore, the isolation and maintenance of primary adult SCs is a crucial step required to address their functions in vitro.

To date, various methods have been developed for primary SC isolation, such as enzymatic digestion [2], sometimes followed by fluorescence-activated cell sorting (FACS) [3,4]. However, these methods have limitations such as the toxicity of fluorochromes (e.g., propidium iodide) [2] and the possible harmful effects of the laser beam used in FACS [5]. Similarly, for enzymatic digestion, a high number of animals is needed and the harsh enzymatic treatment results often in low yields and a damaged cell surface [2,6,7,8,9]. All of these protocols for the isolation of primary SCs have similar problems such as repeated sacrifice of animals, lack of purity, a decline in hormone responsiveness after few passages, and most importantly, the cells have a limited life span in culture. 

Since primary SCs usually reach senescence after few population doublings, thus immortalization is often used. However, immortalization with the SV40 virus T antigen or other methods [10] might result in the partial gain of mesenchymal characteristics, which somehow impacts the suitability of immortalized SCs [11]. Therefore, an easy and reproducible method for the isolation of highly pure, viable, and long-term functional primary SCs is of paramount importance. Recently, conditional reprogramming (CR) was successfully established for the long-term cultivation of primary epithelial cells, with only very few changes of cell characteristics and maintenance of cell lineage commitment [12,13]. The CR method involves the co-culture of irradiated mouse fibroblasts (feeder cells) with primary epithelial cells in the presence of the ROCK inhibitor Y-27632 [14,15]. The irradiated feeder cells can also be replaced by a conditioned medium (CM) generated from them [15]. Originally, Y-27632 was described to promote the viability of human keratinocytes [16] and in feeder cell/keratinocytes co-cultures [17], where it induced cell reprogramming and caused the cells to indefinitely proliferate [18]. As demonstrated previously with keratinocytes, removal of the CM restored the cell capacity to differentiate [15]. Taken together, CR is now a well-established method for long-term propagation of primary epithelial cells without significant alterations of epithelial cell characteristics. 

The analysis of hormone responsiveness and formation of tight junctions (TJs) as an equivalent of the BTB are the key attributes of SC function to study in vitro [19,20], which relate to their substantial roles in spermatogenesis and immune privilege [21,22]. Sertoli cells are highly responsive to many hormones, with androgens and the follicle-stimulating hormone (FSH) as the most relevant one [20], acting via the androgen receptor (AR), Zrt- and Irt-like protein 9 (ZIP9) [23], and the FSH receptor [24]. Previous studies have clearly shown that the analyses of hormone receptor functions have been hampered by the decline of AR expression in both primary and immortalized SCs [11,25,26].

Therefore, it was the goal of the present investigation to overcome the limitations arising from the isolation and culture of primary SCs to isolate adult rat SCs, which can be maintained for a long period of time by conditional reprogramming without changes in their phenotype or expression of SC-specific genes. The new method described here will possibly enable and facilitate new innovative experiments concerning the involvement of SCs in the formation and maintenance of the BTB, in spermatogenesis, in immune privilege, and be of general relevance to studies of male fertility.

## 2. Material and Methods

The study was approved by the local committee on the Ethics of Animal Experiments of the Justus Liebig University (permit number: M_695Giessen, Germany) and all the experiments were performed in accordance with relevant guidelines and regulations. 

### 2.1. Isolation of Adult Rat SC 

Adult male Sprague Dawley rats ((Crl:CD (SD)IGS; Charles River, Cologne, Germany) weighing 150–200 g were anesthetized with 5% isoflurane (Abbott, Wetzlar, Germany). Testes were removed, briefly rinsed in 70% ethanol, washed with sterile Dulbecco’s phosphate buffered saline (DPBS; Gibco/Thermo Fisher, Frankfurt, Germany), and placed in Dulbecco Modified Eagle Medium/Nutrient Mixture F-12 (DMEM/F-12; Gibco). After removal of the tunica albuginea, the tubules of one testis were disaggregated without rupturing them, transferred to enzymatic solution 1 [10 mL DPBS and 1.5 mg/10 mL collagenase type I (Sigma-Aldrich, Taufkirchen, Germany)], and agitated for 3 min in a shaking water bath at 35 °C (120 oscillations/min). The suspension was allowed to settle by unit gravity for 5 min at room temperature (RT) and the supernatant, containing mostly Leydig cells (LCs), carefully aspirated. The tubules were rinsed gently thrice with 10 mL DPBS and incubated for 15 min in enzymatic solution 2 [10 mL DPBS with 0.5 mg/mL collagenase, 0.5 mg/mL hyaluronidase, and 0.2 mg/mL deoxyribonuclease I (DNASE I), all from Sigma-Aldrich] in a shaking water bath at 35 °C (120 oscillations/min) to dislodge peritubular cells (PCs) from the tubules until they were free from the surrounding tissue, but still intact. Then, 15 mL DPBS was added and the tubules were allowed to settle. The supernatant enriched in PCs was collected and centrifuged (500× *g*, 10 min). The cell pellet was suspended in complete DMEM/F-12, seeded into a T75 (~5 × 10^6^ cells/flask; TPP), and maintained in a humidified incubator (37 °C, 5% CO_2_). After 3 days, the PCs reached confluency and were used for further experiments. 

The tubules were transferred to 2 mL DPBS, added slowly on top of 38 mL 5% Percoll (in DPBS; Sigma-Aldrich) in a 50 mL centrifuge tube, and allowed to settle for 20–30 min at room temperature (RT). After discarding the top 35 mL Percoll, the tubules were washed 3 times with DPBS and incubated in enzymatic solution 3 [10 mL DPBS with 1 mg/mL trypsin (Pan-Biotech, Aidenbach, Germany) and 0.2 mg/mL DNase I] for 15 min in a shaking water bath at 35 °C (120 oscillations/min). The tubules were shaken vigorously every 3 min by hand for 5 s until complete digestion, which was stopped by adding 3 mL 100% fetal calf serum (FCS; Gibco). The SC-enriched tubule suspension was transferred to a new 50 mL centrifuge tube containing 25 mL complete DMEM/F-12 [10% FCS, 1% streptomycin/penicillin (P/S; Thermo Fisher), 1% insulin transferrin-selenium (ITS; Thermo Fisher)] to decrease the viscosity prior to filtration through 100-µm cell strainers (Corning/Greiner, Frickenhausen, Germany). The filters were inverted over a 50 mL centrifuge tube and collected by washing with complete DMEM/F-12, then centrifuged (500× *g*, 5 min, RT). After washing with DPBS twice, the cells were resuspended in 10–15 mL complete DMEM/F12 and cell viability was examined by trypan blue staining using a TC10 cell counter (Bio Rad). Approximately 18–36 × 10^6^ cells were obtained from one testis of one adult rat.

### 2.2. Irradiation of Feeder Cells and Preparation of Conditioned Medium (CM)

The 3T3-J2 mouse fibroblasts were cultured as advised by the manufacturer (Kerafast) in a humidified incubator (37 °C, 5% CO_2_). Complete DMEM (+2 mM L-glutamine (Gibco), 10% bovine calf serum (BCS, GE Healthcare/Thermo Fisher), and 1% P/S) was replaced every 2 days until the cells reached 60–80% confluency in a T175 flask (TPP). The cells were washed with DPBS, incubated with TrypLE (Gibco) for 5 min at 37 °C until detachment, and filled up with 10 mL F medium [375 mL complete DMEM/F-12 and 125 mL of F12 nutrient mix (Gibco)]. The cell suspension (1.0–2.5 × 10^6^ cells/mL) was irradiated with a total dose of 30 Gy (=3000 rad) with a Synergy Elekta 6-MV photon linear accelerator, cultured in a T175 flask (7.0 × 10^6^ cells/30 mL F medium), and incubated at 37 °C and CO_2_. After 72 h, the medium was collected and centrifuged at 300× *g* for 5 min at 4 °C. The supernatant was filtered through 0.22 µm filters (Sigma-Aldrich). For immediate use, one part of fresh F medium was mixed with three parts of CM (filtered supernatant) supplemented with 10 µM ROCK inhibitor (diluted in water; ALX-270–333; Enzo Life Sciences) and 0.1 nM cholera toxin (diluted in water; C8052; Sigma-Aldrich) resulting in complete CM, which can be stored for up to 1 week at 4 °C or up to 6 months at −80 °C.

### 2.3. Culture of Freshly Isolated SC Clusters with CM

Enriched SC clusters (~3.5–5.0 × 10^6^) were suspended in 5 mL complete CM supplemented with 10 µM ROCK inhibitor Y-27632, seeded into a collagen-coated T25 flask and were cultivated in a humidified incubator at 37 °C and 5% CO_2_. Coating was done with 5 mL collagen type I (50 μg/mL, 354236; Corning) in DPBS (in the presence of 1% 1 M acetic acid (Roth) for 1 h in a humidified incubator at 37 °C and 5% CO_2_. After washing with DPBS, the flasks were sterilized with UV light for 1 h before use.

Forty-eight hours after seeding, the medium was discarded and hypotonic shock was performed with 4 mL autoclaved hypotonic solution (20 mM Tris-HCl, pH 7–7.4) for exactly 2 min at RT. After washing with DPBS, 5 mL fresh complete CM was added and cell growth was monitored regularly with a Leica microscope. After 4 days, the SCs were washed with DPBS, detached with 3 mL TrypEL for 3 min at RT, collected, and centrifuged (600× *g*, 3 min). Then, the SCs were seeded on a fresh collagen-coated T25 flask in 5 mL fresh complete CM. The SCs were sub-cultured every 4–5 days and complete CM was replaced every 2 days. After 5–12 days, the SCs reached confluency. 

### 2.4. Immunofluorescence (IF)

Primary adult Sertoli cells (PASC1) were plated at a density of 3.0 × 10^4^ into 24-well-plates (1 mL/well) and incubated for 48 h at 37 °C and 5% CO_2_. After stimulation with 10 nM testosterone (T; Sigma-Aldrich) for 24 h, the cells were rinsed with DPBS and fixed with 100% ice-cold methanol (Roth) on ice for 10 min. The cells were washed with PBS three times for 5 min, and then incubated in a blocking solution (PBS with 3% BSA and 0.3% Triton X-100, both from Sigma-Aldrich) for 1 h at RT. Then, the blocking solution was replaced by a fresh blocking solution containing the primary antibodies at a 1:300 dilution for detection of: AR (SC-7305; Santa Cruz), SOX9 (AB5535; Merck Millipore), alpha smooth muscle actin (ASMA; M0851; DAKO), JAM-3 (AB_2533486, Invitrogen), and ZO-1 (61-7300; Thermo Fisher) and incubated overnight at 4 °C. The cells were washed 3 times with DPBS for 3 min each at RT on an orbital shaker. A fresh blocking solution with the appropriate Alexafluor 488-conjugated donkey anti-rabbit, donkey anti-mouse or Alexafluor 555-conjugated donkey anti-mouse secondary antibody (1:250; A-21206, A-21202 and A-31570, respectively; Life Technologies) was added for 1 h at RT. After washing 3 times with DPBS, the images were obtained using an inverse Olympus IX81 microscope equipped with a fluorescence system.

ImageJ was used for the IF measurements according to the protocol http://www.slu.se/PageFiles/388774/Pacho%-20ImageJ%20measuringcell-fluorescence.pdf, freely available at http://rsbweb.nih.gov/ij/ accessed 12 August 2020. Six cells with surrounding TJs from three independent experiments within or closest to the diagonals of the square optical field were quantified. The values were analyzed with GraphPad Prism5 (GraphPad Software, Inc., San Diego, CA, USA).

### 2.5. RNA Extraction, RT-PCR, and Quantitative Real Time PCR 

For all the qRT-PCR studies, immortalized 93RS2 cells (prepubertal rat SCs without AR expression) [27] and an adult rat testis cDNA were used as controls to ensure the specificity of the primers. Primers were designed with the primer blast software http://www.ncbi.nlm.nih.gov/tools/primer-blast accessed 11 September 2021 and were intron-spanning (Table 1). RNAs were collected directly or from confluent PASC1 cells treated with T. Total RNA was isolated with the RNeasy Mini-Kit (Qiagen, Hilden, Germany) and subjected to the DNase I treatment, as described by the manufacturer. The Reverse Transcription-System first strand cDNA synthesis kit (Invitrogen) was used for cDNA synthesis, as recommended. Briefly, 1 µg cDNA was used in a 25 µL PCR reaction with Taq DNA Polymerase (Bio&Sell, Feucht, Germany) and all the components, as recommended by the manufacturer. After an initial heating at 94 °C for 5 min, 35 cycles were performed as follows: Denaturation at 94 °C for 30 s, annealing at 59 °C for 30 s, and extension at 72 °C for 30 s. A final extension at 72 °C was done for 10 min. 

Real-time PCR amplification was done in duplicates with the iQ^TM^ SYBR Green Super-mix (Bio-Rad) on the iCycler iQ System (Bio-Rad). After an initial heating at 94 °C for 5 min, 40 cycles were performed: Denaturation at 94 °C for 14 s, annealing at 59 °C for 30 s, and extension at 72 °C for 15 s. A final extension at 72 °C was done for 10 min. Gene expression was measured after reaching the ct value and calculated using the Delta—Delta Ct method. GAPDH was used for normalization (Table 1). 

### 2.6. Measurement of Transepithelial Resistance (TER)

The TER measurement was performed as reported [29]. Briefly, 6.0 × 10^4^ PASC1 cells/cm^2^ were seeded on 0.4-μm inserts for 24-well plates (Greiner) and cultured for 48 h until they reached confluency. Then, T (10 nM), IL-6 (200 pg/mL), BMP2 (25 ng/mL) or TGF-β3 (3 ng/mL) were added to the inserts. Controls received only the vehicle. For the TER measurement of PCs, 3.0 × 10^5^ PCs (cells/cm^2^) were seeded on inserts for 2 days. For the co-culture, 6.0 × 10^4^ PASC1 (cells/cm^2^) were seeded on inserts for 2 days, then 2.0 × 10^5^ PCs (cells/cm^2^) were seeded on top of them and treated with the agents mentioned above for 72 h, except for T which lasted 6 days. The counting of days started with the onset of treatment. TER measurements were done with a Millicell ERS-2 epithelial Volt-Ohm meter (Merck Millipore). The Ωxcm^2^ was calculated according to the protocol of the manufacturer and by setting the resistance of cell-free inserts to zero. 

### 2.7. Tracer Diffusion Assay (TDA)

The tracer diffusion assay was performed as published [29]. In brief, PASC1 cells cultured on inserts as described above were treated with IL-6, BMP2 or TGF-β3 for 48 h. Then, cells were incubated with a fresh medium containing 5 mg/mL FITC-coupled Dextran, molecular weight 4 kDa (FD4, Sigma-Aldrich), and loaded into the upper compartment of the inserts. After 4 h, a 100 µL sample was taken from the lower compartment and the fluorescence intensity was measured at 490/520 nm (extinction/emission) in an ELISA reader (Tecan) in black 96-well plates (Greiner). 

### 2.8. Statistical Analysis

All the experiments were repeated independently for at least three times with duplicates. Means and SEM values of all the experiments were used for the analysis. Comparisons of the means between more than two groups were performed by the one-way analysis of variance (ANOVA) followed by Dunnett’s multiple comparison test. Student’s *t*-test was used for comparison of the two groups using the GraphPad Prism software (version 5.0, GraphPad Inc.), *p* ≤ 0.05 was considered significant.

## 3. Results 

### 3.1. Isolation of Adult Rat SCs

To date, SC isolation methods provide primary cells that can be passaged only a few times, thus requiring repeated animal sacrifice. Herein, we describe a new protocol for the isolation and long-term maintenance without the need of repeated animal sacrifice of adult rat SCs, which is based on conditional reprogramming. 

**Figure 1:**
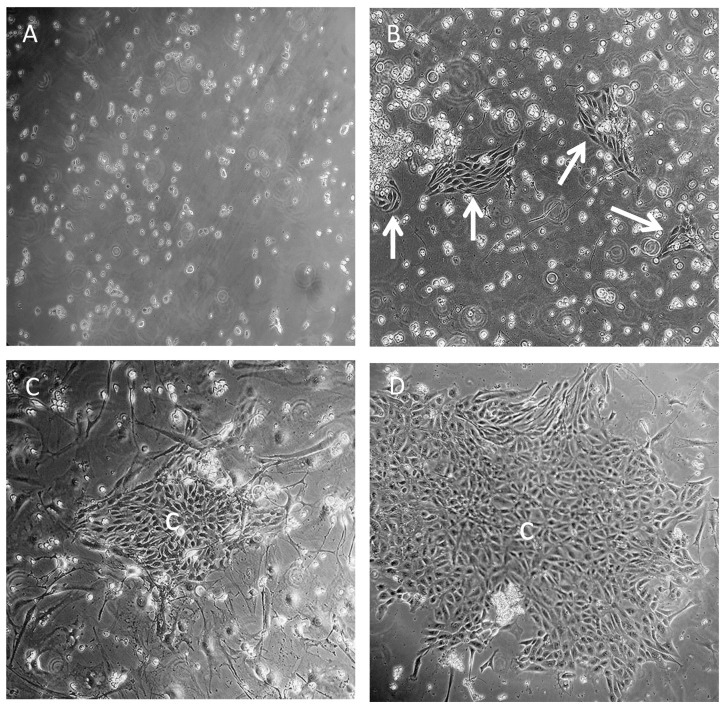
Freshly isolated single SCs (**A**) or SC clusters (**B**–**D**) were seeded on collagen-coated T25 flasks with (**C**) or without irradiated feeder cells (**A**,**B**,**D**). After 24 h (**A**,**B**) or 72 h (**D**) complete CM was added. Single SCs showed less numbers and patches (**A**) compared to SC clusters with larger patches of SCs after 24 h (**B**, arrows). After 72 h, SC clusters with complete CM only (**D**) formed larger colonies (c) compared to SC clusters seeded onto irradiated feeder cells (**C**) (magnification × 100).

**Figure 2:**
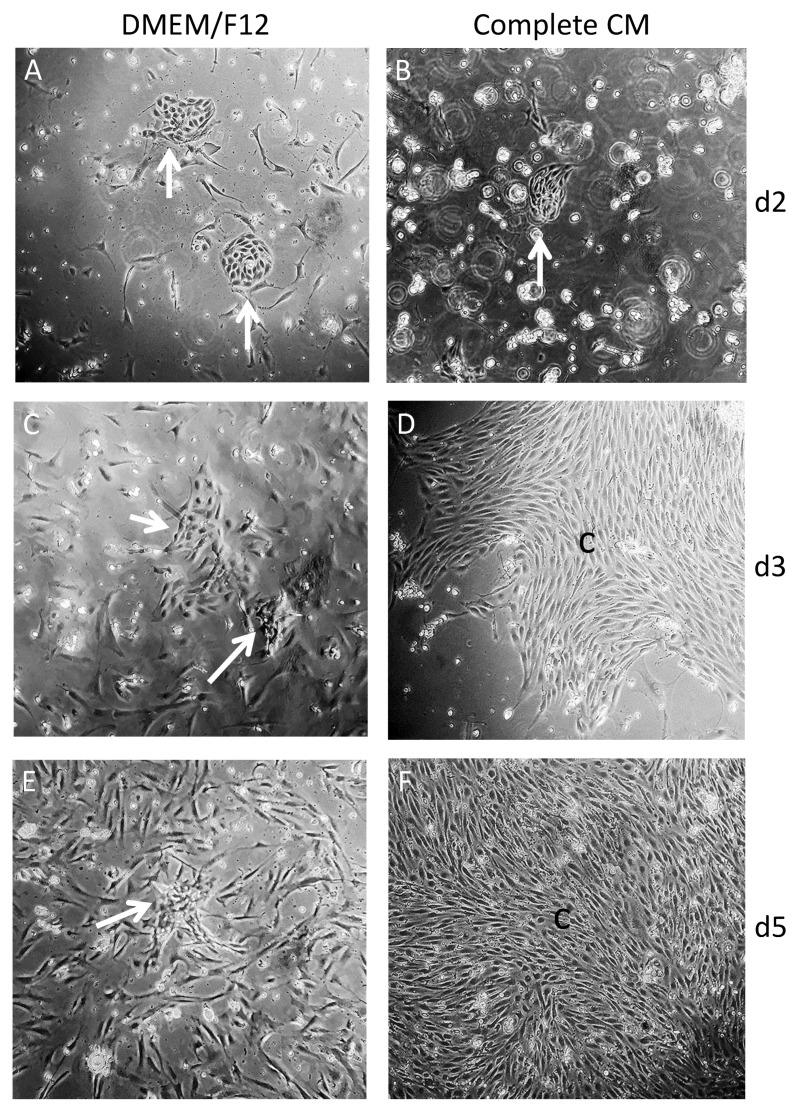
Comparison of freshly isolated SC clusters with complete CM (**B**,**D**,**F**) compared to complete DMEM/F12 (**A**,**C**,**E**) in collagen-coated flasks. At day 2 (d2), SC clusters generated with complete DMEM/F12 compared to complete CM demonstrated no significant differences in the numbers and sizes of patches (**A**,**B**, arrows). However, a higher number of contaminating PCs/LCs is visible in complete DMEM/F12 (**A**) compared to complete CM (**B**), which seems to inhibit the growth of PCs. At day 3 (d3), SC clusters in complete CM formed larger clusters (c) with fewer contaminating PCs/LCs (**D**) compared to SC clusters in complete DMEM/F12 (**C**). At day 5 (d5), contaminating PCs/LCs had overgrown the SC colonies in complete DMEM/F12 (**E**, arrow) in contrast to the confluent SC monolayer (c) in complete CM (**F**) (magnification × 100).

Our first attempt of co-culturing irradiated feeder layers of 3T3-J2 mouse fibroblasts with SCs performed as described [15], showed that the single cells rarely formed colonies typical for SCs (Figure 1A). In contrast, the use of cell strainers with larger pores, 100 µm rather than the previously used 40 µm or 70 µm strainers [30], demonstrated that more presumable SC clusters were attached (Figure 1B). Co-culturing of presumable SC clusters with 3T3-J2 irradiated feeder cells for 3 days resulted in small colonies (Figure 1C). However, the separation of feeder cells from SCs was challenging and contaminating PCs/LCs were phenotypically nearly indistinguishable from the irradiated feeder cells (Figure 1C). In contrast, the use of conditioned medium rather than an irradiated feeder layer showed incomplete CM and complete DMEM/F12 small presumable SC clusters, as early as 2 days after seeding (Figure 2A,B). After 3 days, larger presumable SC clusters in complete CM compared to those in complete DMEM/F12 could be observed (Figure 2C,D). A microscopic examination showed the disappearance of presumable SC clusters in DMEM/F12 (Figure 2E). In contrast, complete CM supported presumable SC clusters to reach near confluency (Figure 2F). Of note, compared to complete DMEM/F12 (Figure 2C,E), the complete CM inhibited the growth of non-epithelial testicular cells after 5 days (Figure 2D,F).

**Figure 3:**
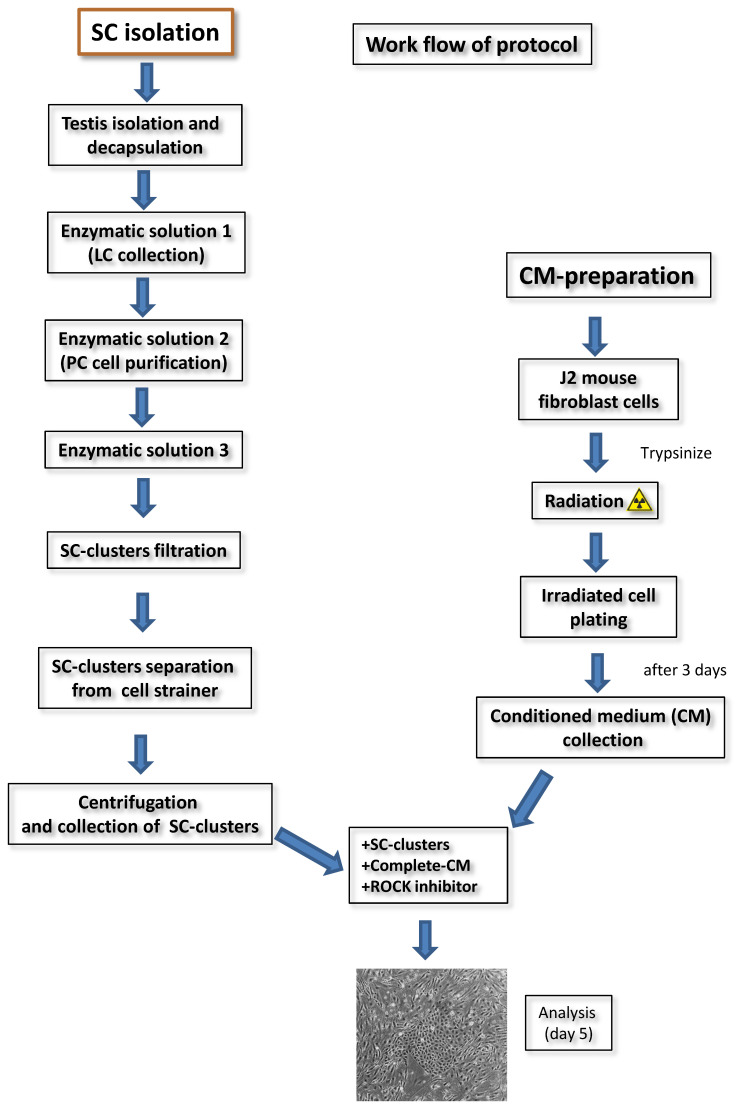
An overview of the CR method for the isolation of adult rat SCs is given.

All in all, with complete CM, the growth of SCs was supported and much easier to monitor due to the lack of contaminating feeder cells, thus CM is preferable to feeder cells. The modified and optimized protocol based on Liu et al. [15] is summarized in Figure 3. To date, we generated three independent, conditionally reprogrammed adult rat SCs populations with a doubling time of ~20 h, which have been termed primary adult rat SCs 1–3 (PASC1–3). The following data exemplarily present the characterization of PASC1, which has now undergone 210 doublings in 175 days and reached passage 31. Conditionally reprogrammed SCs can be easily frozen and thawed without any problems, with cell viability at least ≥93%. 

### 3.2. Characterization of PASC1—Expression of Testicular Markers

**Figure 4:**
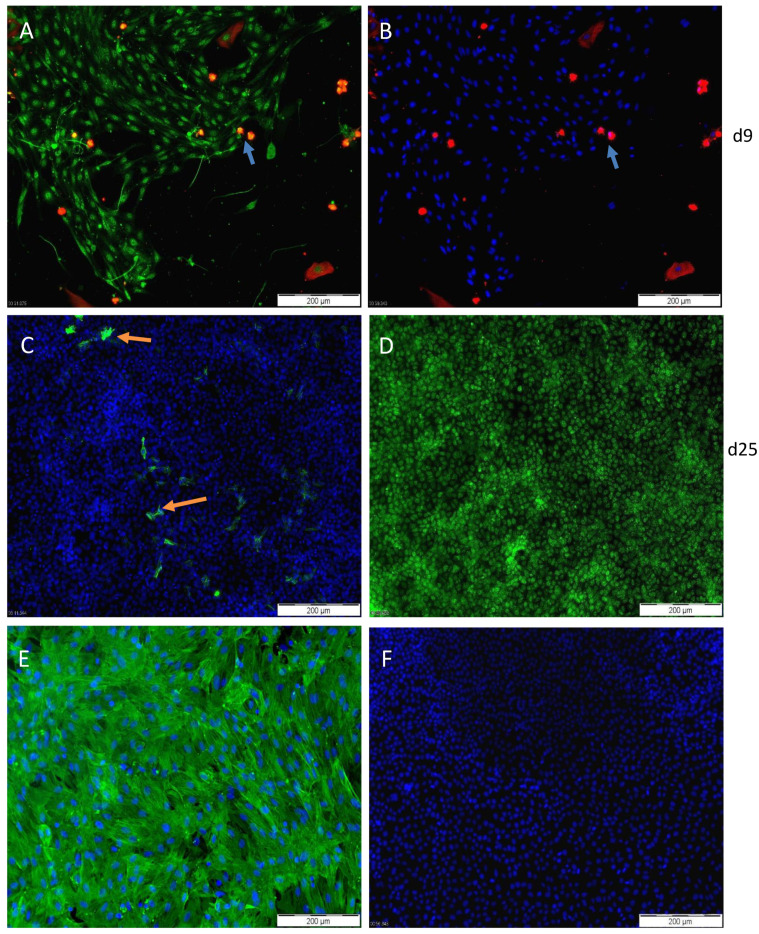
PASC1 were cultivated in complete CM for 9 (**A**,**B**), and 25 days (**C**,**D**), and primary PCs for 18 days (**E**) then stained with antibodies against SOX9 (**A**,**D**, green) and ASMA (**A**,**B**, red, **C**,**E** green). At day 9 (d9), PASC1 positive for SOX9 (green) are shown in addition to very few contaminating ASMA-positive PCs (red) (**A**,**B**, arrows indicating few PCs); the round phenotype indicates that the PCs are dying due to the complete CM, as published [14,15]. At day 25 (d25), the purity of SCs is increased, with only very few ASMA-positive PCs (**C**, arrows, green) with ~99% of the cells positive for SOX9 (**D**, green). Primary PCs stained with ASMA were used as a positive control (**E**); in (**F**), the negative control without any primary antibody is shown. The nuclei were stained with DAPI (blue).

**Figure 5:**
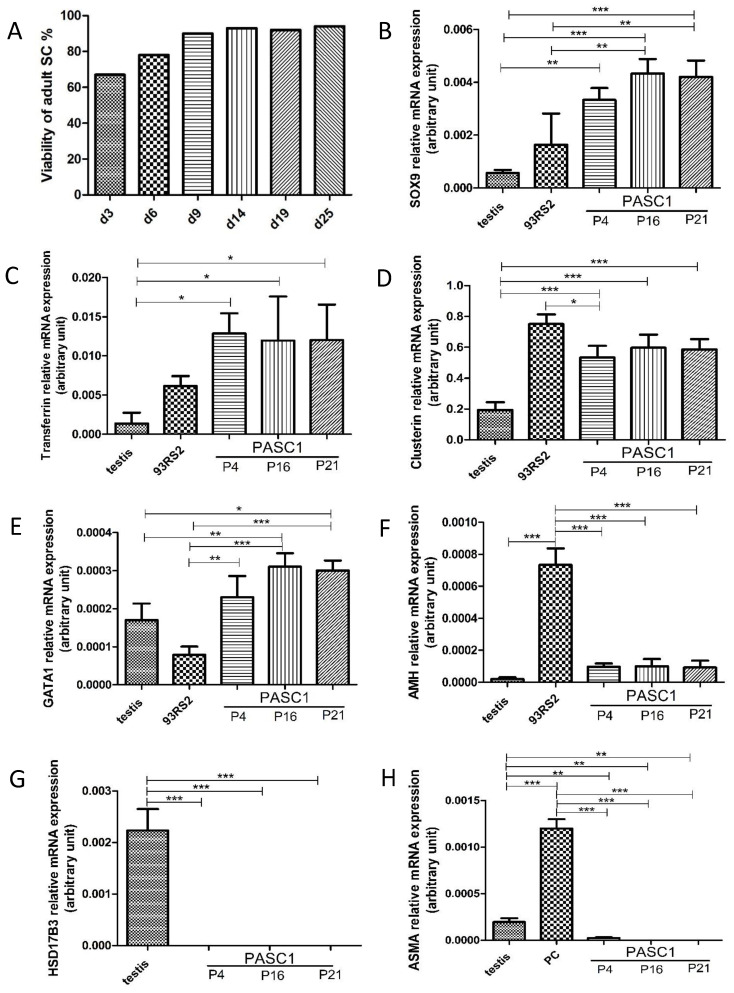
The viability of PASC1 increased strongly after 9 days (d9) of isolation from 63.5% at day 3 to 93% at day 25 (**A**). Gene expression of SC-specific and SC-maturation genes in passages 4 (P4), 16 (P16), and 21 (P21) was monitored with qRT-PCR. Immortalized immature rat 93RS2 SCs and adult rat testis were used as positive controls. In addition to a strong expression of SOX9 (**B**), transferrin (**C**), clusterin (**D**), and GATA1 (**E**), PASC1 showed a weak expression of AMH, a marker of immature SCs (**F**). Contamination of PASC1 by LCs was excluded using HSD17B3 (**G**) and by ASMA for PCs (**H**). Each bar represents the mean ± SEMs of three independent experiments performed in duplicates; * *p* ≤ 0.05; ** *p* < 0.01; *** *p* < 0.001.

To address the PASC1 purity after isolation, IF staining with the SC-specific marker SOX9 and the PC-specific marker ASMA showed that nearly all the PASC1 cells expressed SOX9 and not ASMA resulting in a purity of ~99% (Figure 4). Of note, viability improved significantly after 6–9 days and was maintained over time (Figure 5A). The qRT-PCR was used to further examine SC-specific and SC-maturation genes (Table 1) and demonstrated that PASC1 cells express SC-specific SOX9, transferrin, and clusterin (Figure 5B–D) [31,32,33]. GATA1, a post-pubertal SC maturation gene [34,35], is strongly expressed in PASC1 compared to 93RS2 indicating that PASC1 cells maintained their differentiated state even without CM (Figure 5E). In contrast, the Anti-Müllerian hormone (AMH), a marker of prepubertal SCs [36], is only very weakly expressed in PASC1 compared to the immature 93RS2 cells (Figure 5F). One of the hallmarks of conditional reprogramming, in addition to the long-term viability of epithelial cells, is the selective growth of epithelial cells, whereas the non-epithelial cells die in the first passages [14,15]. As shown in Figure 5G, LCs were no longer detectable in passage 4, whereas very small amounts of ASMA-positive PCs were found, which were no longer detectable in the later passages (Figure 5H). 

### 3.3. Functional Characterization of PASC1—Formation of the SC Barrier 

To test whether PASC1 are a suitable model with which to study the adult SC function in vitro, the expression of TJs and formation of the TJ barrier were analyzed as a proof of principle.

**Figure 6:**
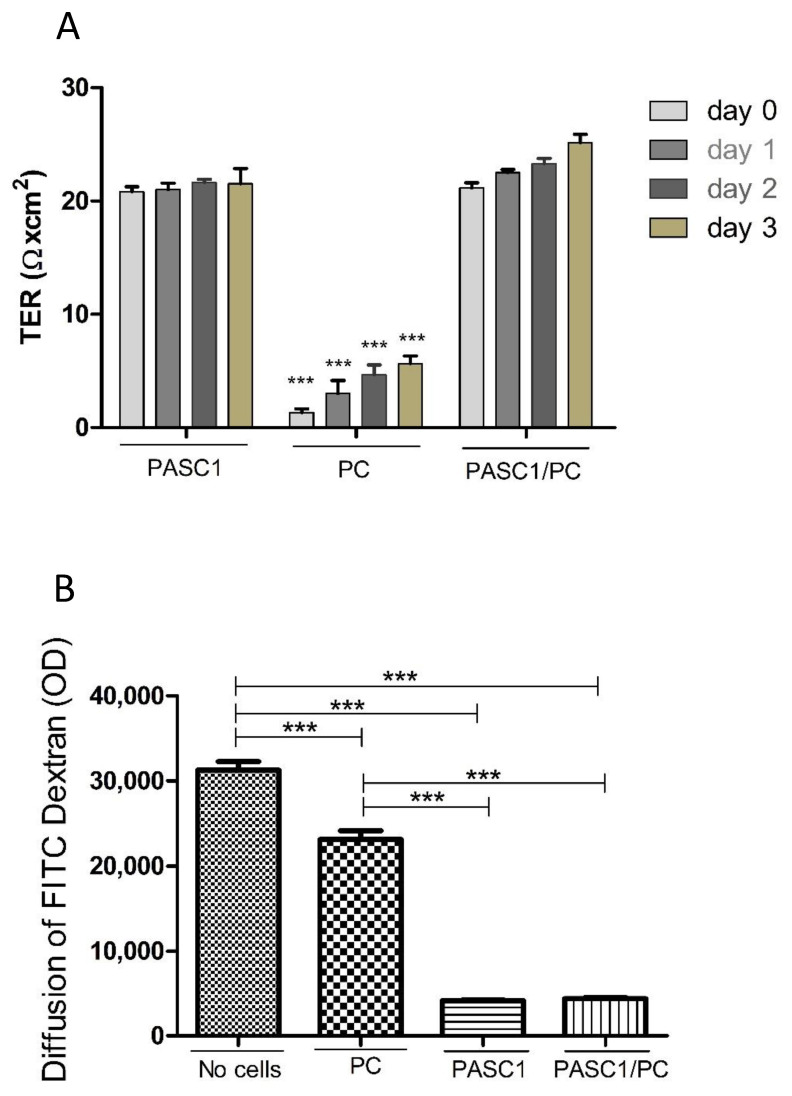
Time-dependent effects of different single cell types or co-cultures on barrier integrity. Monocultures of PASC1 showed a strong barrier, which was only slightly improved by co-cultures of PASC1/PCs, as shown by the TER values (**A**). Statistical tests were performed to evaluate differences between the distinct days and the distinct mono- and co-cultures; for example, day 0 showed a significant reduction only for PC compared to PASC1, and for the PASC1/PC co-culture, but the difference between PASC1 and PASC1/PC was not significant. Monocultures of PASC1 showed the strongest reduction of the diffused FITC-dextran after 48 h compared to PCs only (**B**). Co-cultures of PASC1/PCs only showed slightly higher reductions compared to mono-cultures of PASC1. Data points represent the mean values ± SEMs obtained from three independent repetitions performed in duplicates (n = 6); *** *p* < 0.001.

Moreover, we aimed to elucidate the contribution of PCs to the TJ barrier. For this purpose, PASC1 or PCs were seeded on cell culture inserts individually or in co-cultures until confluency was achieved. The results showed that PASC1 cells contributed mainly to the TJ barrier, whereas PCs alone only formed a very weak barrier (Figure 6A), similarly to co-cultures, where PCs only contributed slightly to the barrier (Figure 6A). In addition, the tracer diffusion assay (TDA) also showed that in contrast to PCs and co-cultures, mainly PASC1 contributed to the TJ barrier (Figure 6B).

**Figure 7:**
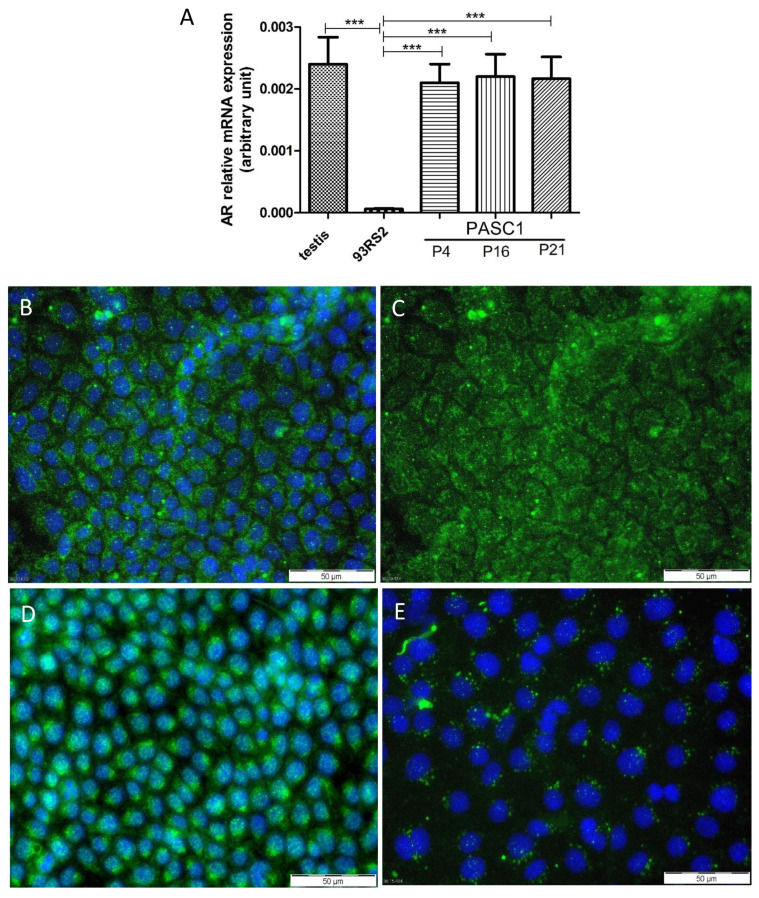
Conditionally reprogrammed PASC1 express the androgen receptor (AR) on the mRNA (**A**) and protein level (**B**–**D**) in passages P4 and P16. The mRNAs of 93RS2 and adult rat testis were used as negative and positive controls, respectively. The majority of AR (green) was detected in the cytoplasm of PASC1 and rarely in the nuclei (**B**,**C**) of untreated cells. After the treatment with testosterone, the AR was visible in the nuclei and concentrated in a perinuclear region (**D**). The negative control with Alexa Fluor showed a negligible background (**E**). DAPI was used to stain the nuclei (blue). Each bar represents the mean ± SEMs of three independent experiments performed in duplicates; *** *p* < 0.001.

The formation of TJs at the BTB is hormone-dependent [23,37], thus, AR expression and hormone responsiveness were investigated. PASC1 showed a strong AR mRNA and protein expression in the three different passages, P4, P16, and P21 (Figure 7). Moreover, PASC1 indicate SC maturation [38] and that AR expression is maintained in PASC1 cells even after long-term maintenance in vitro. Furthermore, they showed a strong expression of ZIP9-specific mRNA in RT-PCR (data not shown), which has been established in recent experiments as an androgen receptor of physiological significance [39,40]. The AR was found in untreated cells mainly in the cytoplasm (Figure 7B,C), but localized to the nucleus and a perinuclear region after testosterone stimulation (Figure 7D). 

### 3.4. Effects of T on the PASC1 Barrier Integrity

**Figure 8:**
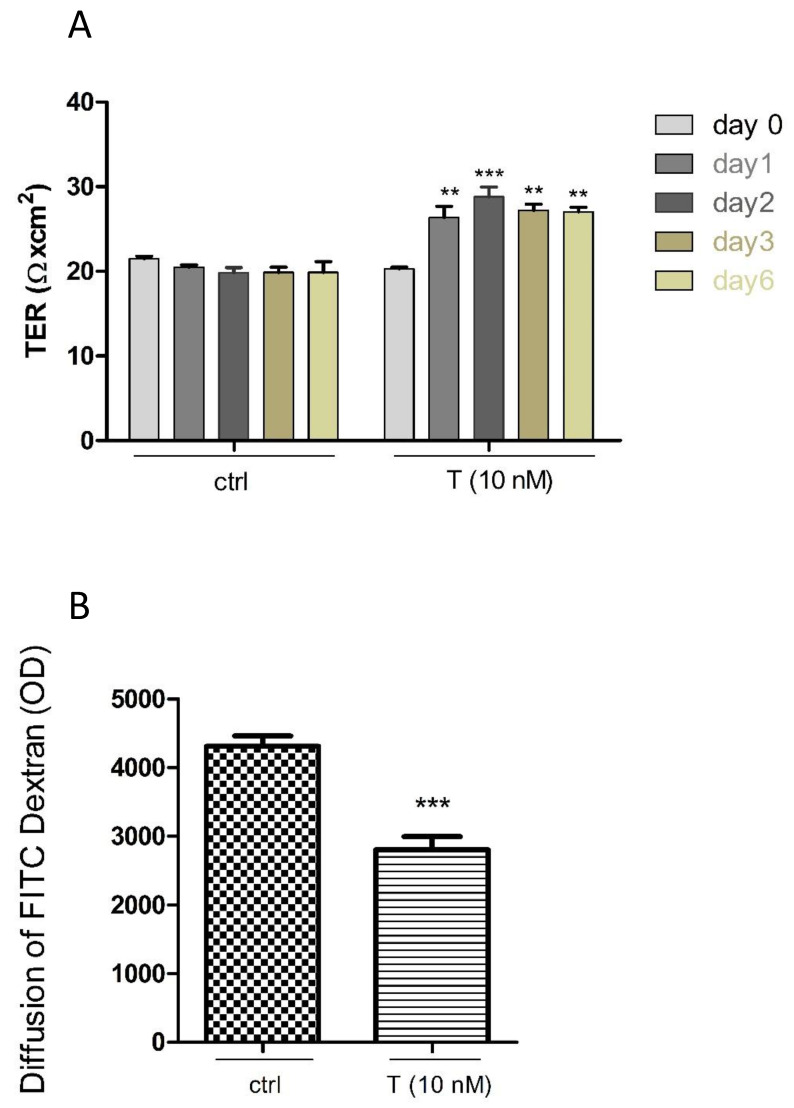
Time-dependent effects of testosterone (T) on the PASC1 TJ barrier. T increased the TJ integrity in a time-dependent manner as shown by the increased TER values compared to the untreated controls (ctrl) (**A**). Stimulation of PASC1 with T for 48 h resulted in a significant reduction of diffusion of FITC-dextran (**B**). Data points represent the mean values ± SEMs obtained from three independent repetitions performed in duplicates (n = 6); ** *p* < 0.01; *** *p* < 0.001 (**A**) and the unpaired *t*-test was used for statistical analysis; *** *p* < 0.001 (**B**). OD: Optical density.

In order to prove the effects of androgens on the SC barrier, PASC1 on cell culture inserts were treated with T. As clearly shown in Figure 8A, T enhances the TER values of PASC1 in a time-dependent manner compared to the controls. Furthermore, TDA showed that T decreased the permeability (Figure 8B), indicating that T promoted the TJ integrity of PASC1 in vitro.

**Figure 9:**
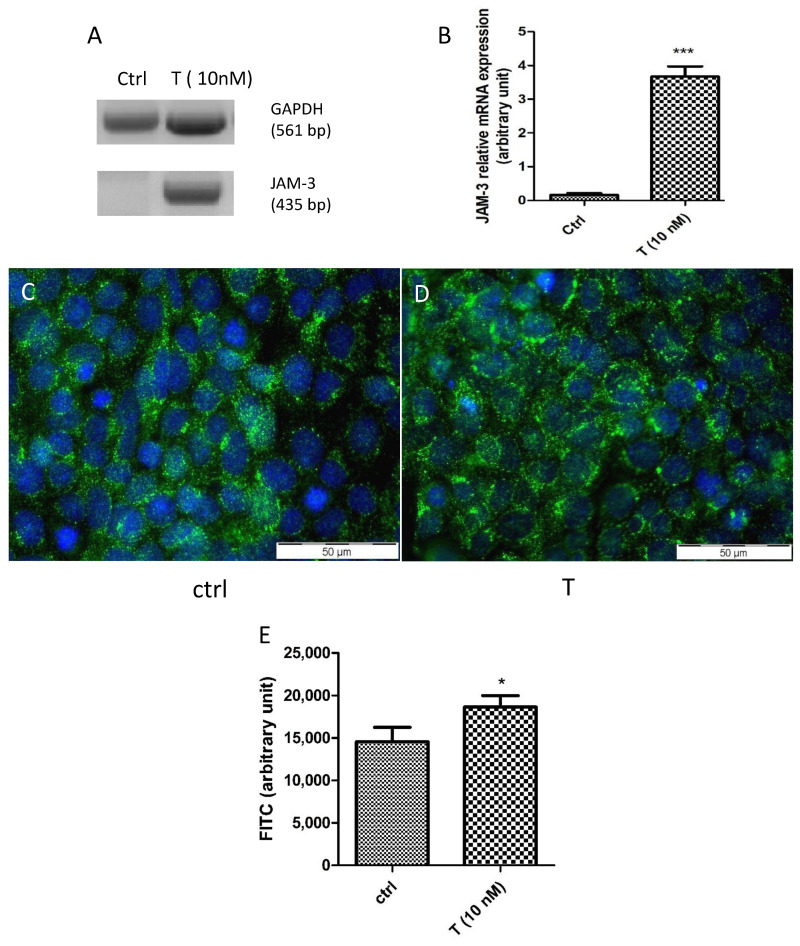
Effects of testosterone (T) on JAM-3 mRNA expression (**A**,**B**) and protein presence (**C**–**E**). PASC1 (2 × 10^5^ cells/well) were treated with T for 24 h. The JAM-3 mRNA expression was strongly increased in PASC1 after the T treatment compared to the control (ctrl) (**A**,**B**). JAM-3 is localized mainly in the cell membrane in a punctate pattern but can be also found in the cytoplasm (**C**). T stimulated the JAM-3 protein expression strongly (**D**) and significantly (**E**). DAPI was used to stain the nuclei (blue). Each bar represents the mean ± SEMs of three independent experiments performed in duplicates. The unpaired *t*-test was used for statistical analysis; * *p* ≤ 0.05; *** *p* < 0.001.

**Figure 10:**
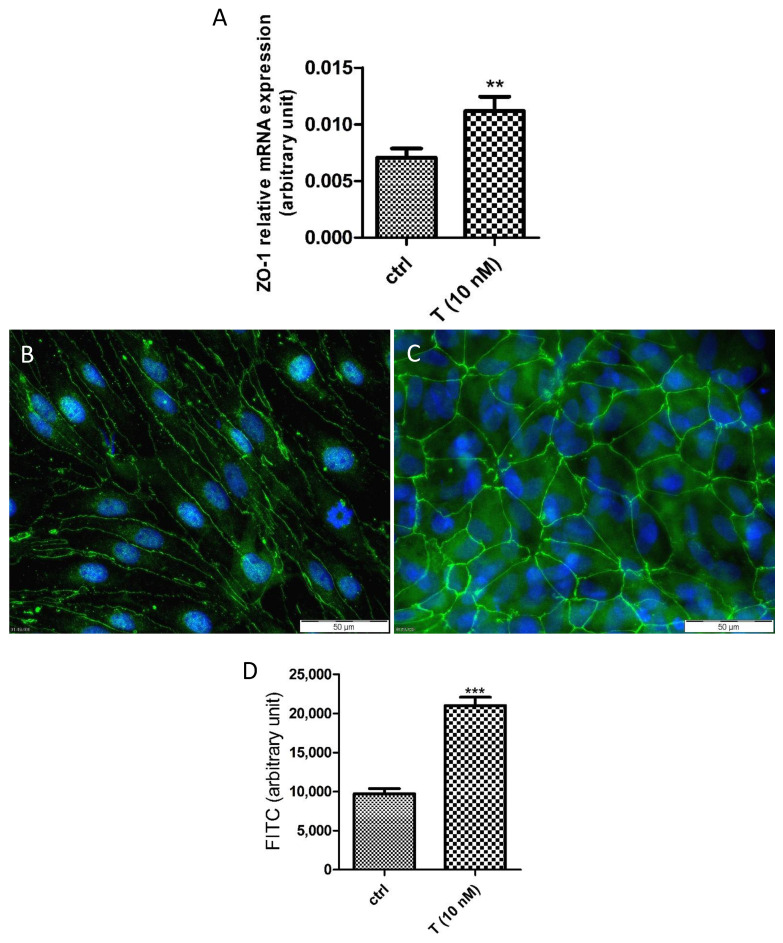
Effects of testosterone (T) on ZO-1 mRNA expression (**A**) and protein presence (**B**–**D**). PASC1 (2 × 10^5^ cells/well) were treated with T for 24 h. The ZO-1 mRNA expression was significantly increased by the T treatment (**A**) and compared to the untreated cells (**B**). A stronger protein presence was observed between cell-to-cell contacts in T-treated cells (**C**). Quantification showed a significant increase (**D**). DAPI was used to stain the nuclei (blue). Each bar represents the mean ± SEMs of three independent experiments performed in duplicates. The unpaired *t*-test was used for statistical analysis; ** *p* < 0.01; *** *p* < 0.001.

The TJ proteins JAM-3 and ZO-1 are involved in testicular cell-to-cell contacts [41] and both are androgen-dependent [23,42,43,44]. The treatment of PASC1 with T strongly upregulated the JAM-3 mRNA expression (Figure 9A,B) as well as the cytoplasmic and cell membrane associated JAM-3 protein significantly (Figure 9C–E). Similarly, the T treatment also upregulated the ZO-1 mRNA expression (Figure 10A) and ZO-1 protein abundance (Figure 10B–D) at cell-to-cell contacts significantly compared to the untreated controls. Of note, the T treatment changed the cell morphology slightly from more elongated (Figure 10B) to more cobblestone-like (Figure 10C).

### 3.5. Effects of Different Cytokines on the TJ Barrier of PASC1

**Figure 11:**
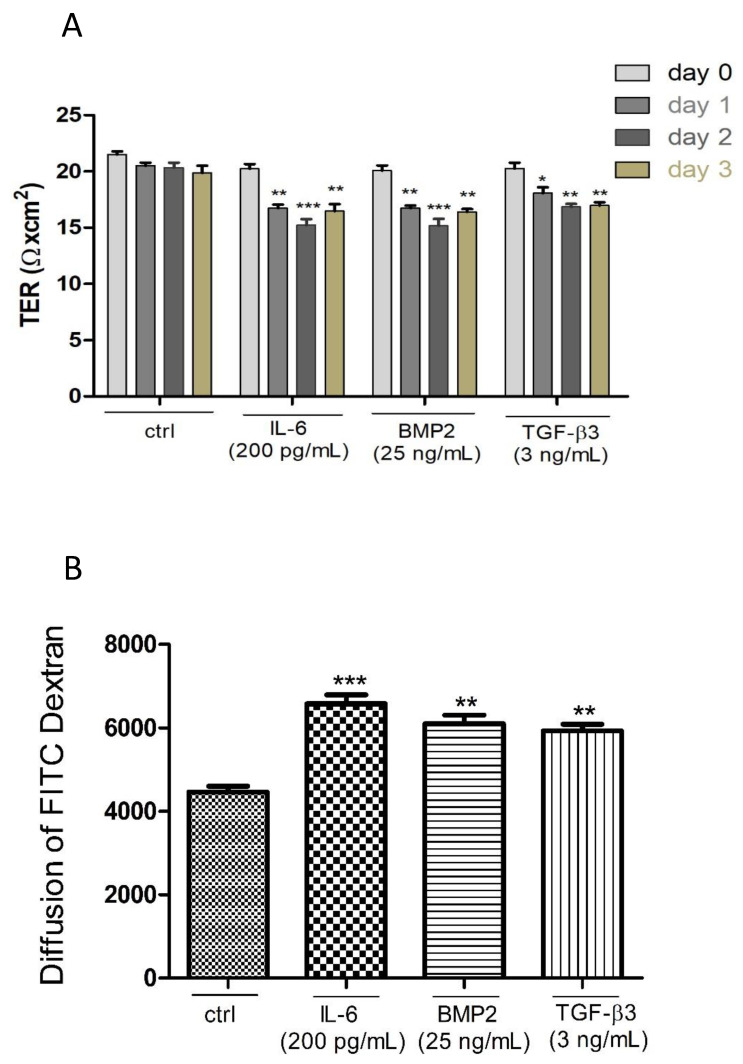
Treating PASC1 with IL-6, BMP2 or TGF-β3 decreased the TER values time-dependently as compared to the untreated controls (ctrl) (**A**). After the treatment of PASC1 with IL-6, BMP2 or TGF-β3 for 48 h, a significant increase of diffusion of FITC-dextran was observed. The values are normalized to the control (=100) (**B**). Data points represent the mean values ± SEMs obtained from three independent repetitions performed in duplicates (n = 6); * *p* ≤ 0.05; ** *p* < 0.01; *** *p* < 0.001. OD: Optical density.

Some testicular cytokines have a strong impact on the BTB [45]. Therefore, we used interleukin-6 (IL-6) [46] and the transforming growth factor beta-3 (TGF-β3) [47,48] as gold standards, in addition to the bone morphogenetic protein-2 (BMP2) [49]. PASC1 on cell culture inserts were treated with the three cytokines for 24–72 h. The integrity of the TJ barrier was downregulated especially after 48 h by each substance compared to the untreated controls (Figure 11A). TDA measurements confirmed the TER results (Figure 11B).

## 4. Discussion

Although significant advancements occurred in SC biology, our understanding of adult SCs especially in rodents is severely hampered by the lack of protocols for long-term propagation. In this study, we established a robust and reproducible new protocol for the isolation and long-term maintenance of adult rat SCs based on conditional reprogramming [14,15]. The conditionally reprogrammed PASCs were highly pure, viable, and demonstrated similar morphological and functional characteristics as SCs in vivo. Compared to the previous methods [50], the execution of the protocol described here requires the sacrifice of one animal only and is more efficient in terms of the enzymes used (~20-fold lower trypsin concentration) compared to the other reports [6,9]. Additionally, the time consumption for the isolation of primary adult SCs is ~2 h versus ~6–8 h. Although other authors also described the fast and successful isolation of adult SCs [7,50,51], the method described here generates higher yields, possibly since we used SC clusters rather than single SCs. On the other hand, we also observed that it is better to avoid any disturbance of the tubules especially in the beginning, confirming the observations by Anway et al. [7]. As suggested by Liu et al. [15], we tried the conditional reprogramming of SCs with irradiated feeder cells or a complete CM, but found the latter to be preferable. However, it is important that either the irradiated 3T3-J2 mouse fibroblasts or conditioned medium generated from these cells is necessary to get rid of the fibroblasts, and thus to have highly pure epithelial cells. Therefore, to date, we have established three viable adult SC cell lines of high purity and could maintain them also over a long period of time in vitro.

As exemplarily shown for one SC cell line, PASC1, they were nearly 100% pure and express the SC-specific protein SOX9 as well as the epithelial genes transferrin and clusterin, which are characteristic for SCs [31,32,33]. Similarly, as shown for epithelial cells from other organs such as breast, prostate, colon, and lungs [15,51,52], the conditionally reprogrammed PASC1 maintained their differentiation even after passage 21 by expressing the SC-maturation gene GATA1 in contrast to a very weak expression of AMH, a typical marker for immature SCs [34,36]. Moreover, we could observe that the non-epithelial cells such as LCs and PCs are lost in the early passages as reported for non-epithelial cells from other tissues [14,15]. This is a very important advantage, since especially PCs are the major contaminating cells in primary SCs and influence SC functions [1,9]. Therefore, the highly pure PASC1 cells are now the first primary SCs, which can be studied without PC/LC contaminations. In contrast to the immortalization methods such as SV-40 large T antigen or hTERT, which have been reported to cause cell alterations and loss of cell characteristics over time [10,11,53], PASC1 maintained their differentiation state even after long-term maintenance. As a proof of principle, we performed the analysis of androgen-responsive genes in PASC1 and the formation of the TJ barrier as an in vitro proxy of the BTB. 

To the best of our knowledge, we could show for the first time that adult SCs form a tight barrier by TJs with only a negligible contribution by PCs. These results are in accordance with immature SCs/PCs co-cultures [54,55]. Barrier integrity is also promoted by androgens [19]. Compared to other cell lines, such as, for example, epididymal epithelial cells, which form a very tight barrier [29], SCs show only low/moderate TER/TDA values. However, it is conceivable that other factors contribute to the BTB, since the cause(s) of the testicular immune privilege are still unknown [22]. 

Our results showed convincingly that PASC1 maintained a high level of the AR gene and protein expression in early and late passages. Furthermore, responsiveness was also functional as demonstrated by the increased mRNA and protein expression of ZO-1 and JAM-3 and the increased integrity of TJs after the androgen treatment. Of note, preliminary experiments indicate that androgen responsiveness is also maintained in later passages (data not shown). This is a clear difference to primary SCs, which most often loose the AR expression and function after a few passages in vitro [26]. The expression of ZO-1 and JAM-3 proteins is androgen-dependent and their mRNA expression was observed to begin around puberty [42,44]. Remarkably, ZO-1 was recently reported to associate with occludin, and thus to contribute to the integrity of the BTB [56]. The involvement of ZO-1 and JAM3 in cell-to-cell contacts and their androgen-dependent expression are supported by our findings. However, which androgen receptor is the mediator of the effects described here still needs to be addressed. PASC1, next to the classical AR, also express ZIP9, a newly established membrane-bound androgen receptor of physiological and pathophysiological significance [23,39,40]. In 93RS2 rat Sertoli cells lacking AR, ZIP9 is the receptor that mediates testosterone signaling leading to the expression of TJ-forming proteins [23]. 

Many cytokines are expressed in the testis and some of them have been described to be involved in the regulation of the BTB [57]. We have chosen IL-6, TGF-β3, and BMP2, three cytokines well-known to be expressed in the testis, and IL-6 and TGF-β3 have been described to perturb the integrity of the BTB between immature rat SCs in vitro [45,46,47]. As shown for immature SCs, we also found that IL-6 and TGF-β3 reduced the TJ barrier in adult PASC1. To date, BMP2 has been shown to be mainly implicated in immature spermatogonial cells and SC signaling and proliferation [58,59]. Interestingly, our results showed for the first time that BMP2 negatively affected the TJ integrity in adult SCs. The involvement of BMP2 in the regulation of the blood testis barrier is further supported by findings such as the disturbance of the intestinal mucosal barrier via occludin [60] or of the epithelial barrier in the lung by BMP2 [61]. 

## 5. Conclusions

We have established a new and reliable method to isolate SCs from a limited amount of tissue of adult rat testis. Conditional reprogramming with conditioned media resulted in a high yield, high purity, and long-term maintenance of adult SCs without dedifferentiation. The newly generated PASC1 cell line has proven to be an ideal model to study androgen dependency and the TJ formation of adult rat SCs in vitro. Although other hormones and SC functions still need to be investigated, our new protocol will be helpful in reducing animal sacrifices and to enable new innovative studies with adult Sertoli cells.

## Figures and Tables

**Table 1:** List of primer sequences used for RT-PCR or qRT-PCR.

Genes (Species) and Acc. No.	Sequence (5′ -> 3′)	AT	Size (bp)
GAPDH (rat) XM_039107008.1	GACCCCTTCATTGACCTCAAC fwd GATGACCTTGCCCACAGCCTT rev	59 °C	561
ASMA (rat) NM_031004.2	GAAGAGGAAGACAGCACAGC fwd TTGGTGATGATGCCGTGTTC rev	58 °C	233
SOX9 (rat) [28] NM_080403.2	CATCAAGACGGAGCAACTGAG fwd GTGGTCGGTGTAGTCATACTGC rev	59 °C	148
Transferrin [11] NM_001013110.1	TATTGGCCCAGCAAAATGTG fwd CCGGAACAAACAGAAATTGC rev	59 °C	370
Clusterin (rat) [11] XM_039092999.1	AGGAGCTAAACGACTCGCT fwd GCTTTTCCTGCGGTATTCC rev	59 °C	362
GATA1 (rat) NM_012764.2	ATAGCAAGACGGCGCTCTAC fwd CACTCTCTGGCCTCACAAGG rev	59 °C	319
AMH (rat) NM_012902.1	AACTGACCAATACCAGGGGC fwd GGCTCCCATATCACTTCAGCC rev	59 °C	334
AR (rat) [11] NM_012502.2	GCCAGTGGCTGAGGATGAG fwd GGTGAGCTGGTAGAAGCGC rev	59 °C	236
JAM-3 (rat) NM_001004269.1	CTTCTTCCTGCTGCTGCTCT fwd TCTTGGCATTGCAGTGTTGC rev	59 °C	435
HSD17B3 (rat) NM_054007	GGAAGCCGTGTGAAGGTT fwd GACACTCTGGCTCTCACC rev	58 °C	171
ZO-1 (rat) XM_0391053461	CTTGCCACACTGTGACCCTA fwdGGGGCATGCTCACTAACCTT rev	58 °C	262

AT: Annealing temperature; Acc. No.: Accession number; fwd: Forward; rev: Reverse; bp: Base pairs; GAPDH: Glyceraldehyde 3-phosphate dehydrogenase; ASMA: Alpha smooth muscle actin; HSD17B3: Hydroxysteroid 17-beta dehydrogenase 3; AMH: Anti-Mullerian Hormone; AR: Androgen Receptor; JAM-3: Junctional Adhesion Molecule-3; ZO-1: Zonula occludens-1.

## Data Availability

Not applicable.
